# Supplementary material for: Tolerability of oral itraconazole and voriconazole for the treatment of chronic pulmonary aspergillosis: A systematic review and meta-analysis
Source: PLoS One. 2020 Oct 14;15(10):e0240374. doi: 10.1371/journal.pone.0240374 (PMC7556473; doi:10.1371/journal.pone.0240374)
Supplement: S1 Fig — (DOCX) [file pone.0240374.s001.docx]

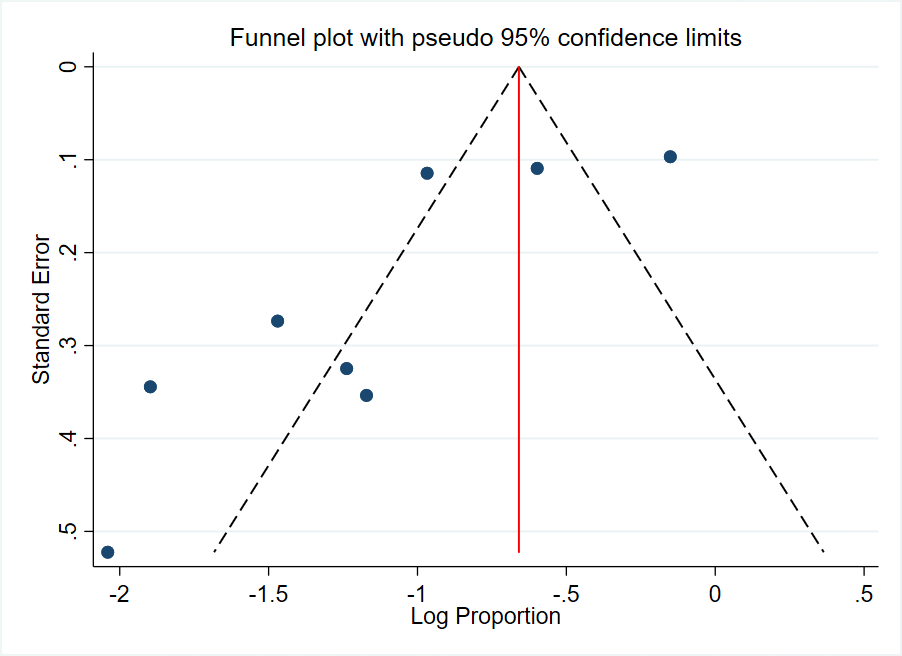


**Supplementary file 3.** Distribution of studies reporting AEs to voriconazole. Note the asymmetry of the funnel plot possibly due to the small sample size of the included studies and moderate degree of publication bias.
