# Supplementary figures and images for: Tolerability of oral itraconazole and voriconazole for the treatment of chronic pulmonary aspergillosis: A systematic review and meta-analysis
Source: PLoS One. 2020 Oct 14;15(10):e0240374. doi: 10.1371/journal.pone.0240374 (PMC7556473; doi:10.1371/journal.pone.0240374)

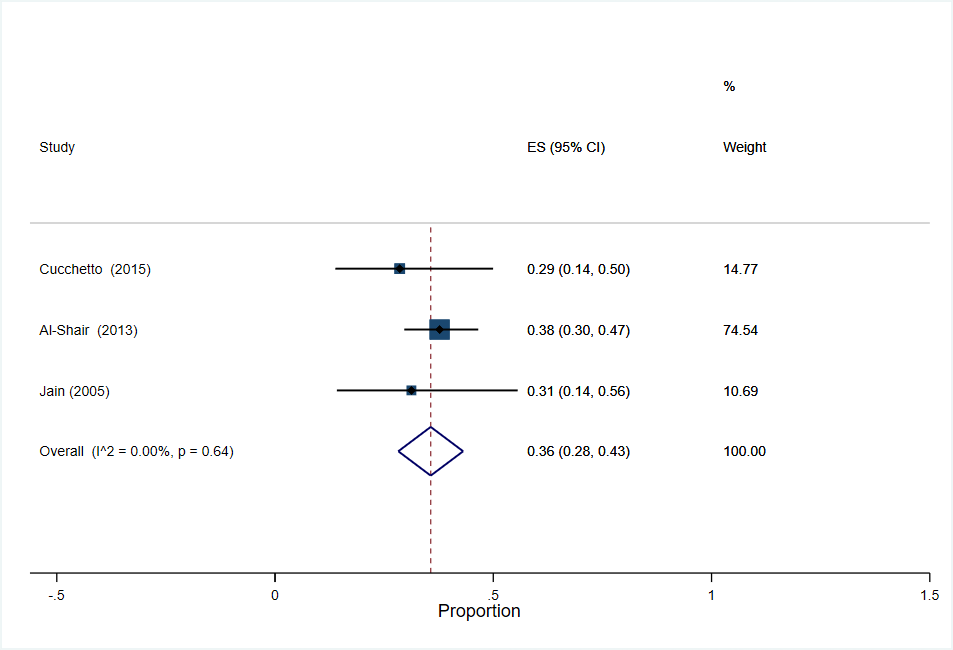

Supplement: S2 Fig — (PNG) [file pone.0240374.s002.png]
