# Supplementary material for: Tolerability of oral itraconazole and voriconazole for the treatment of chronic pulmonary aspergillosis: A systematic review and meta-analysis
Source: PLoS One. 2020 Oct 14;15(10):e0240374. doi: 10.1371/journal.pone.0240374 (PMC7556473; doi:10.1371/journal.pone.0240374)
Supplement: S2 File — (DOCX) [file pone.0240374.s004.docx]

12 May 20 - 09:37

HDAS Export

Strategy Felix Bongomin - adverse events to Itraconazole and voriconazole - May 2020

[See full search strategy](#historyanchor)

Strategy 850709/saved

Contents 15 of 15 results on Saved Results

[1. Assessment of posaconazole salvage therapy in chronic pulmonary aspergillosis using predefined response criteria.](#f8f7e3ee-d63d-6fdc-3f12-14d9758ad9df-1)

[2. Isavuconazole and voriconazole for the treatment of chronic pulmonary aspergillosis: A retrospective comparison of rates of adverse events.](#83f27bff-7581-ef02-7b6c-1b12db368ea1-2)

[3. Twelve-month clinical outcomes of 206 patients with chronic pulmonary aspergillosis.](#ec550c46-aa26-f645-8142-242ef17929d0-3)

[4. Voriconazole for chronic pulmonary aspergillosis: a prospective multicenter trial.](#173c49a1-d0ac-7e8f-1c2e-fbf3970b773b-4)

[5. Treatment of chronic pulmonary aspergillosis by voriconazole in nonimmunocompromised patients.](#8c2e22af-5de0-aaf0-0f8d-4e6566a5658e-5)

[6. Treatment of chronic pulmonary aspergillosis with voriconazole: review of a case series.](#b630a588-6073-2474-afd1-f16b80a32bcc-6)

[7. Efficacy and safety of voriconazole in the treatment of chronic pulmonary aspergillosis: experience in Japan.](#ff0e722d-0bed-4820-9a48-fc6e3b55725e-7)

[8. Therapeutic drug monitoring and adverse events of delayed-release posaconazole tablets in patients with chronic pulmonary aspergillosis](#b89eee51-b855-282e-f1b4-31d3f23bc979-8)

[9. A cross-sectional survey to measure the prevalence of chronic pulmonary aspergillosis (CPA) complicating pulmonary tuberculosis in Northern Uganda](#84e2adf7-83f6-e01f-8e3b-0617fec0607e-9)

[10. The Management of Chronic Pulmonary Aspergillosis: The UK National Aspergillosis Centre Approach](#02c8663d-637e-f8f7-ab42-af99b187b1cf-10)

[11. Impact of liposomal amphotericin B therapy on chronic pulmonary aspergillosis](#5f8b1386-ad52-e7ac-6e21-3fa5d439980f-11)

[12. Long-term antifungal treatment improves health status in patients with chronic pulmonary aspergillosis: A longitudinal analysis](#ae4848f0-97dd-2982-278c-a35311829364-12)

[13. The correlation between serum trough levels and frequency of side effects of VRCZ at Nagasaki University Hospital, Nagasaki Japan](#ff7011fe-7f87-2a49-c6c8-2e4773243507-13)

[14. Chronic pulmonary aspergillosis (CPA): Role of <sup>18</sup>F -FDG PET/CT](#3b4e3f03-38d3-adbd-f09b-8f423d420765-14)

[15. Efficacy and safety of posaconazole for chronic pulmonary aspergillosis](#3dc0f724-30b7-e077-a33d-fd9ea3b150f1-15)

[Full strategy](#historyanchor)

Results Saved Results

**15** of **15 saved results**

**1. Assessment of posaconazole salvage therapy in chronic pulmonary aspergillosis using predefined response criteria.**

**Author(s):** Rodriguez-Goncer, Isabel; Harris, Chris; Kosmidis, Chris; Muldoon, Eavan G; Newton, Pippa J; Denning, David W

**Source:** International journal of antimicrobial agents; Aug 2018; vol. 52 (no. 2); p. 258-264

**Publication Date:** Aug 2018

**Publication Type(s):** Journal Article

**PubMedID:** 29906567

Available at [International journal of antimicrobial agents](https://www.research.manchester.ac.uk/portal/files/75750947/Posaconazole_Rodriguez_Goncer_2018.pdf) - from Unpaywall

**Abstract:**OBJECTIVESChronic pulmonary aspergillosis (CPA) is a progressive infection that destroys lung tissue in non-immunocompromised patients. First-line therapies for CPA (itraconazole and/or voriconazole) are often curtailed due to toxicity or the development of drug resistance. Posaconazole is a potential alternative for these patients.METHODSUse of posaconazole was funded by the National Health Service Highly Specialised National Commissioners on an individual basis for patients who failed or did not tolerate first-line therapy; those who met predefined criteria for improvement at 4 and 6 months (weight gain and/or improvement in St George's Respiratory Questionnaire) continued posaconazole long-term. We recorded response, failure, discontinuation rates, and adverse events.RESULTSSeventy-eight patients received posaconazole as salvage therapy. Thirty-four (44%) achieved targets for continuation of therapy. Fourteen (18%) failed therapy; five (36%) patients did not achieve clinical targets at 4 or 6 months of assessment and nine (64%) developed clinical and/or radiological failure. Twenty-eight (36%) discontinued their trial early; 8 (29%) died and 20 (71%) had significant side effects. One patient was non-compliant and another was lost to follow up.CONCLUSIONSEstablishing criteria for therapeutic success offered a clear, safe and sustainable method of identifying patients who benefit from additional therapy, and minimised continuation of ineffective therapy in those who did not.

**Database:** Medline

**2. Isavuconazole and voriconazole for the treatment of chronic pulmonary aspergillosis: A retrospective comparison of rates of adverse events.**

**Author(s):** Bongomin, Felix; Maguire, Niamh; Moore, Caroline B; Felton, Timothy; Rautemaa-Richardson, Riina

**Source:** Mycoses; Mar 2019; vol. 62 (no. 3); p. 217-222

**Publication Date:** Mar 2019

**Publication Type(s):** Comparative Study Journal Article Observational Study

**PubMedID:** 30570179

Available at [Mycoses](https://go.openathens.net/redirector/nhs?url=https%3A%2F%2Fonlinelibrary.wiley.com%2Fdoi%2Ffull%2F10.1111%2Fmyc.12885) - from Wiley Online Library Medicine and Nursing Collection 2019 - NHS

Available at [Mycoses](http://search.ebscohost.com/login.aspx?direct=true&scope=site&site=ehost-live&db=mdc&AN=30570179) - from EBSCO (MEDLINE Complete)

Available at [Mycoses](http://openurl.ebscohost.com/linksvc/linking.aspx?genre=article&issn=0933-7407&volume=62&issue=3&spage=217) - from EBSCO (Biomedical Reference Collection - Comprehensive)

Available at [Mycoses](https://www.research.manchester.ac.uk/portal/files/83891363/Bongomin_et_al_2018_Mycoses.pdf) - from Unpaywall

**Abstract:**BACKGROUNDLong-term oral triazole antifungal therapy is the cornerstone of management for patients with chronic pulmonary aspergillosis (CPA). Itraconazole is the first-line choice of treatment. Voriconazole, posaconazole or isavuconazole can be used as alternative treatments in case of resistance or intolerance. All of these can cause significant adverse drug reactions.OBJECTIVESTo evaluate how CPA patients tolerate voriconazole and isavuconazole after prior triazole therapy.METHODSWe performed a retrospective observational study at the UK National Aspergillosis Centre. Medical records for all consecutive CPA patients started on isavuconazole and voriconazole during an observation period of 12 and 6 months respectively were analysed.RESULTSDuring this study period, 20 patients were started on isavuconazole and 21 patients on voriconazole. Adverse events were seen in 18 of 21 (86%) the patients in the voriconazole group and 12 of 20 (60%) in the isavuconazole group (P = 0.02). For those who developed adverse events to these agents, the rates of discontinuation of therapy were comparable (ie 10/18 [56%], voriconazole vs 8/12 [67%], isavuconazole; P = 0.54). Five (25%) patients in the isavuconazole group who were intolerant to other triazoles tolerated the standard dose of isavuconazole.CONCLUSIONSCompared with isavuconazole, adverse events were significantly higher in CPA patients commenced on voriconazole. Isavuconazole may be an option for those patients who are intolerant to other triazoles.

**Database:** Medline

**3. Twelve-month clinical outcomes of 206 patients with chronic pulmonary aspergillosis.**

**Author(s):** Bongomin, Felix; Harris, Chris; Hayes, Gemma; Kosmidis, Chris; Denning, David W

**Source:** PloS one; 2018; vol. 13 (no. 4); p. e0193732

**Publication Date:** 2018

**Publication Type(s):** Research Support, Non-u.s. Gov't Journal Article

**PubMedID:** 29634721

Available at [PloS one](https://dx.plos.org/10.1371/journal.pone.0193732) - from Public Library of Science (PLoS)

Available at [PloS one](http://search.ebscohost.com/login.aspx?direct=true&scope=site&site=ehost-live&db=mdc&AN=29634721) - from EBSCO (MEDLINE Complete)

Available at [PloS one](http://gateway.proquest.com/openurl?ctx_ver=Z39.88-2004&res_id=xri:pqm&req_dat=xri:pqil:pq_clntid=48092&rft_val_fmt=ori/fmt:kev:mtx:journal&genre=article&issn=1932-6203&volume=13&issue=4&spage=e0193732) - from ProQuest (Health Research Premium) - NHS Version

Available at [PloS one](https://journals.plos.org/plosone/article/file?id=10.1371/journal.pone.0193732&type=printable) - from Unpaywall

**Abstract:**There is a paucity of evidence surrounding the optimal antifungal therapy for use in chronic pulmonary aspergillosis (CPA) and the duration of therapy remains unclear. We retrospectively evaluated treatment outcomes, including change in quality of life scores (St George's Respiratory Questionnaire (QoL)), weight and Aspergillus IgG at 6 and 12 months following initiation of therapy in a cohort of 206 CPA patients referred to the UK National Aspergillosis Centre (NAC), Manchester between April 2013 and March 2015. One hundred and forty-two patients (69%) were azole naïve at presentation and 105 (74%) (Group A) were commenced on itraconazole, 27 (19%) on voriconazole, and 10 (7%) were not treated medically. The remainder (64 patients, 31%) had previously trialled, or remained on, azole therapy at inclusion (Group B) of whom 46 (72%) received itraconazole, 16 (25%) voriconazole, and 2 (3%) posaconazole. Initial therapy was continued for 12 months in 78 patients (48%) of those treated; the azole was changed in 62 (32%) patients and discontinued in 56 (29%) patients for adverse reactions (32, 57%), azole resistance (11, 20%), clinical failure (8, 14%) or clinical stability (5, 9%). Azole discontinuation rates were higher in Group B than in Group A (42% vs. 22%, p = 0.003). For all patients who survived, weight increased (median of 62.2Kg at baseline, to 64.8 at 12 months), mean Aspergillus IgG declined from 260 (baseline) to 154 (12 months) and QoL improved from 62.2/100 (baseline) to 57.2/100 (12 months). At 12 months, there was no difference in median survival between Groups A and B (95% vs. 91%, p = 0.173). The rate of emergence of resistance during therapy was 13% for itraconazole compared to 5% for voriconazole. Bronchial artery embolization was done in 9 (4.4%) patients and lobectomy in 7 (3.2%). The optimal duration of azole therapy in CPA is undetermined due to the absence of evidenced based endpoints allowing clinical trials to be undertaken. However we have demonstrated itraconazole and voriconazole are modestly effective for CPA, especially if given for 12 months, but fewer than 50% of patients manage this duration. This suggests extended therapy may be required for demonstrable clinical improvement.

**Database:** Medline

**4. Voriconazole for chronic pulmonary aspergillosis: a prospective multicenter trial.**

**Author(s):** Cadranel, J; Philippe, B; Hennequin, C; Bergeron, A; Bergot, E; Bourdin, A; Cottin, V; Jeanfaivre, T; Godet, C; Pineau, M; Germaud, P

**Source:** European journal of clinical microbiology & infectious diseases : official publication of the European Society of Clinical Microbiology; Nov 2012; vol. 31 (no. 11); p. 3231-3239

**Publication Date:** Nov 2012

**Publication Type(s):** Research Support, Non-u.s. Gov't Clinical Trial Multicenter Study Journal Article

**PubMedID:** 22782438

Available at [European journal of clinical microbiology & infectious diseases : official publication of the European Society of Clinical Microbiology](http://search.ebscohost.com/login.aspx?direct=true&scope=site&site=ehost-live&db=mdc&AN=22782438) - from EBSCO (MEDLINE Complete)

Available at [European journal of clinical microbiology & infectious diseases : official publication of the European Society of Clinical Microbiology](http://gateway.proquest.com/openurl?ctx_ver=Z39.88-2004&res_id=xri:pqm&req_dat=xri:pqil:pq_clntid=48092&rft_val_fmt=ori/fmt:kev:mtx:journal&genre=article&issn=0934-9723&volume=31&issue=11&spage=3231) - from ProQuest (Health Research Premium) - NHS Version

Available at [European journal of clinical microbiology & infectious diseases : official publication of the European Society of Clinical Microbiology](https://link.springer.com/content/pdf/10.1007%2Fs10096-012-1690-y.pdf) - from Unpaywall

**Abstract:**Early evidence suggests the efficacy of voriconazole for chronic pulmonary aspergillosis (CPA). We conducted a prospective, open, multicenter trial to evaluate the efficacy and safety of voriconazole for proven CPA in minimally or non-immunocompromised patients. Patients had CPA confirmed by chest computed tomography (CT) and/or endoscopy, positive Aspergillus culture from a respiratory sample, and positive serologic test for Aspergillus precipitins. Patients received voriconazole (200 mg twice daily) for a period of 6-12 months and were followed for 6 months after the end of therapy (EOT). The primary endpoint was global success at 6 months, defined as complete or partial (≥50 % improvement) radiological response and mycological eradication. Forty-one patients with confirmed CPA were enrolled. All patients had A. fumigatus as the etiologic agent. By EOT, five patients had died from comorbidities and seven had discontinued voriconazole due to toxicity. The global success rate at 6 months was 13/41 (32 %): 10/19 (53 %) for chronic necrotizing aspergillosis and 3/22 (14 %) for chronic cavitary aspergillosis (p = 0.01). The respective success rates at EOT were 58 and 32 %. Clinical symptoms and quality of life also improved during treatment. Voriconazole is effective for CPA, with acceptable toxicity. The response rate is higher and obtained more rapidly in necrotizing than cavitary forms.

**Database:** Medline

**5. Treatment of chronic pulmonary aspergillosis by voriconazole in nonimmunocompromised patients.**

**Author(s):** Camuset, Juliette; Nunes, Hilario; Dombret, Marie-Christine; Bergeron, Anne; Henno, Priscilla; Philippe, Bruno; Dauriat, Gaelle; Mangiapan, Gilles; Rabbat, Antoine; Cadranel, Jacques

**Source:** Chest; May 2007; vol. 131 (no. 5); p. 1435-1441

**Publication Date:** May 2007

**Publication Type(s):** Clinical Trial Multicenter Study Journal Article

**PubMedID:** 17400661

Available at [Chest](https://linkinghub.elsevier.com/retrieve/pii/S0012369215316135) - from American College of Chest Physicians (ACCP)

Available at [Chest](https://linkinghub.elsevier.com/retrieve/pii/S0012369215316135) - from Geneva Foundation for Medical Education and Research (Free Medical Journals)

Available at [Chest](https://mft.nhs.uk/medical-education/trust-library/locations-opening-hours-contact-us/) - from Oxford Road Campus Print Holdings Local Print Collection

**Abstract:**BACKGROUNDThere is no recognized medical treatment for chronic pulmonary aspergillosis (CPA) apart from surgery in patients with simple aspergilloma. To evaluate the efficacy of voriconazole in this setting, we conducted a retrospective multicenter study over a 3-year period.METHODSFor inclusion in the study, patients had to have received voriconazole for treatment of confirmed or probable CPA with a follow-up of at least 6 months. Clinical, radiologic, and mycologic data were collected at baseline, every 2 to 3 months, and at the end of treatment or at the date point.RESULTSTwenty-four patients were included in the study, among which 9 patients presented with chronic cavitary pulmonary aspergillosis and 15 presented with chronic necrotizing pulmonary aspergillosis (CNPA). Voriconazole was given as a first-line treatment to 13 patients. The median duration of treatment and follow-up were 6.5 and 10 months, respectively. Three patients had to stop treatment with voriconazole because of toxicity. Symptoms and imagery findings were improved in 16 of 24 patients and 17 of 24 patients, respectively, at the end of follow-up. Mycology, which was positive at baseline in 21 of 23 patients, was negative in 18 of 19 patients at the end of follow-up; serologic test results were also negative in 6 of 19 evaluable patients, all of whom had CNPA. Improved radioclinical findings and mycologic eradication were observed at the end of follow-up in 11 of 19 patients (58%). Patients in whom the disease was controlled had a significantly longer median duration of treatment than patients in whom it was uncontrolled (9 vs 6 months, respectively; p = 0.04).CONCLUSIONVoriconazole provides effective treatment of CPA with an acceptable level of toxicity.

**Database:** Medline

**6. Treatment of chronic pulmonary aspergillosis with voriconazole: review of a case series.**

**Author(s):** Cucchetto, G; Cazzadori, A; Conti, M; Cascio, G L; Braggio, P; Concia, E

**Source:** Infection; Jun 2015; vol. 43 (no. 3); p. 277-286

**Publication Date:** Jun 2015

**Publication Type(s):** Research Support, Non-u.s. Gov't Journal Article Observational Study

**PubMedID:** 25432571

Available at [Infection](http://search.ebscohost.com/login.aspx?direct=true&scope=site&site=ehost-live&db=mdc&AN=25432571) - from EBSCO (MEDLINE Complete)

Available at [Infection](http://gateway.proquest.com/openurl?ctx_ver=Z39.88-2004&res_id=xri:pqm&req_dat=xri:pqil:pq_clntid=48092&rft_val_fmt=ori/fmt:kev:mtx:journal&genre=article&issn=0300-8126&volume=43&issue=3&spage=277) - from ProQuest (Health Research Premium) - NHS Version

**Abstract:**PURPOSEChronic pulmonary aspergillosis (CPA) is a rare disease that primarily affects subjects with moderate immunodepression and/or structural alterations in the lung.METHODSData for patients with probable CPA were collected over 24 months. Patients with probable CPA received oral voriconazole, and clinical, laboratory and radiological follow-up was performed at 3, 6 and 12 months.RESULTS21 patients (mean age 52.4 years) were evaluated. Factors predisposing to CPA were tuberculosis (n = 8), chronic obstructive pulmonary disease (n = 7), corticosteroids (n = 14), chemo- or radio-therapy (n = 6), tracheostomy or endotracheal prosthesis (n = 5), smoking (n = 4), asthma (n = 3), and chronic liver disease (n = 3). Sputum or bronchial aspirate cultures were positive for Aspergillus spp. in 14 cases (66.6 %). (1,3)-β-D-glucan on serum was positive in 16 cases (76.2 %). Excavated pulmonary thickening was evident in 14 patients (66.6 %) and in 9 of these cases (64.2 %) aspergilloma was present. [(18)F]2-fluoro-2-deoxy-D-glucose-PET-CT was positive in 13/15 patients, and simple aspergilloma was diagnosed after surgical excision in one of the negative cases. All patients were treated with oral voriconazole. Therapy was discontinued due to skin toxicity (n = 3), liver toxicity (n = 2) and severe mental disorder (n = 1). At 12 months' follow-up, nine patients (42.9 %) were considered cured or improved. Seven patients (33.3 %) died during follow-up, mainly due to underlying disease.CONCLUSIONSA reasonable proportion of patients achieved cure or improvement with voriconazole, but 28.5 % of treated patients had to discontinue therapy because of toxicity. The high mortality makes it difficult to fully assess the real efficacy of voriconazole and to establish the correct duration of therapy.

**Database:** Medline

**7. Efficacy and safety of voriconazole in the treatment of chronic pulmonary aspergillosis: experience in Japan.**

**Author(s):** Saito, T; Fujiuchi, S; Tao, Y; Sasaki, Y; Ogawa, K; Suzuki, K; Tada, A; Kuba, M; Kato, T; Kawabata, M; Kurashima, A; Sakatani, M; NHO Pulmonary Fungosis Research Group

**Source:** Infection; Dec 2012; vol. 40 (no. 6); p. 661-667

**Publication Date:** Dec 2012

**Publication Type(s):** Multicenter Study Journal Article

**PubMedID:** 22956473

Available at [Infection](http://search.ebscohost.com/login.aspx?direct=true&scope=site&site=ehost-live&db=mdc&AN=22956473) - from EBSCO (MEDLINE Complete)

Available at [Infection](http://gateway.proquest.com/openurl?ctx_ver=Z39.88-2004&res_id=xri:pqm&req_dat=xri:pqil:pq_clntid=48092&rft_val_fmt=ori/fmt:kev:mtx:journal&genre=article&issn=0300-8126&volume=40&issue=6&spage=661) - from ProQuest (Health Research Premium) - NHS Version

**Abstract:**BACKGROUNDThough various clinical conditions of aspergillosis can occur, depending essentially on the host's immunological status, the focus of research in North American and European countries has mainly been on invasive pulmonary aspergillosis in immunocompromised patients. There are, however, also many problems to overcome in chronic forms of aspergillosis. One of those problems is that there are no codified treatment guidelines for chronic pulmonary aspergillosis (CPA). Especially in Japan, this issue is more serious, because there are more cases with CPA due to the many aged people with past history of tuberculosis. Several clinical cases and case series have reported the usefulness of the various antifungal agents that are available. The new triazole, voriconazole, in particular, seems to be effective in the treatment of CPA. The aim of the present study is to evaluate the efficacy and safety of voriconazole in the treatment of CPA in non-immunocompromised patients.PATIENTS AND METHODSWe conducted a prospective, open-label, non-comparative, multicenter study over a 2-year period. For inclusion in the study, patients with confirmed or probable CPA were recruited in 11 hospitals of the National Hospital Organization in Japan. Clinical, radiological, serological, and mycological data were collected at baseline and 12 weeks after treatment or at the end of treatment.RESULTSAmong 77 patients enrolled in the study, 71 patients (mean age 65.9 years, 56 males and 15 females) were eligible for the study. All of the eligible patients presented with underlying lung diseases, including sequelae of tuberculosis (n = 35), non-tuberculous mycobacterial lung disease (n = 8), chronic obstructive pulmonary disease (COPD) (n = 8), interstitial pneumonia (n = 7), cystic lung disease (n = 4), pneumothorax (n = 3), bronchial cancer (n = 1), and others (n = 5). Voriconazole was indicated in 48 cases (68 %) as the first-line treatment for CPA and 23 patients previously received other antifungal therapies. Based on a composite of clinical, radiologic, serological, and mycologic criteria, good response was seen in 43 patients (60.6 %), no response was observed in 19 patients (26.8 %), and 4 cases (5.6 %) got worse. Five patients (7.0 %) were unassessable for efficacy. The common adverse events were visual disturbances (17 patients, 23.9 %), abnormal liver function test results (12 patients, 16.9 %), adverse psychological effects (3 patients, 4.2 %), and others (10 patients, 14.0 %). Treatment with voriconazole had to be stopped in 2 cases (2.8 %) because of serious adverse events (abnormal liver function test results). There was no association between adverse effects and trough voriconazole levels in serum.CONCLUSIONSIn Japan, voriconazole provides effective therapy of CPA in non-immunocompromised patients with an acceptable level of toxicity.

**Database:** Medline

**8. Therapeutic drug monitoring and adverse events of delayed-release posaconazole tablets in patients with chronic pulmonary aspergillosis**

**Author(s):** Kosmidis C.; Rautemaa-Richardson R.; Richardson M.D.; Denning D.W.; Rodriguez-Goncer I.; Moore C.B.

**Source:** Journal of Antimicrobial Chemotherapy; 2019; vol. 74 (no. 4); p. 1056-1061

**Publication Date:** 2019

**Publication Type(s):** Article

**PubMedID:** 30590533

Available at [The Journal of antimicrobial chemotherapy](https://academic.oup.com/jac/article/74/4/1056/5262264) - from Oxford Journals - Medicine

Available at [The Journal of antimicrobial chemotherapy](https://academic.oup.com/jac/article/74/4/1056/5262264) - from HighWire - Free Full Text

Available at [The Journal of antimicrobial chemotherapy](https://academic.oup.com/jac/article/74/4/1056/5262264) - from Geneva Foundation for Medical Education and Research (Free Medical Journals)

Available at [The Journal of antimicrobial chemotherapy](https://www.research.manchester.ac.uk/portal/en/publications/therapeutic-drug-monitoring-and-adverse-events-of-delayedrelease-posaconazole-tablet-in-patients-with-chronic-pulmonary-aspergillosis(b807ac35-ed71-47d2-a59c-bf92024985f7).html) - from Unpaywall

**Abstract:**Background: Posaconazole delayed-release tablets offer better bioavailability than the liquid suspension, but no post-marketing data are available in immunocompetent hosts such as those with chronic pulmonary aspergillosis (CPA). Objective(s): To explore the pharmacokinetics and adverse event (AE) profile of posaconazole tablets in patients with CPA. Method(s): Patients started on posaconazole tablets at the National Aspergillosis Centre (NAC), Manchester, UK between February 2014 and October 2015 were identified from the NAC database and analysed retrospectively. The medical records were reviewed for factors that could affect posaconazole serum levels and the development of AEs. Result(s): Seventy-two patients were included; 50 (69%) were male and the mean age was 48.5+/-12 years. Therapeutic levels (>=1 mg/L) were achieved in 90% of cases on 200mg versus 90% of cases on 300mg daily (P=not significant). Based on multivariate analysis, female sex (P=0.041), a 100mg daily dose (P<0.001), asthma (P=0.01) and bronchiectasis (P=0.001) were associated with subtherapeutic levels. Forty-nine (68%) patients developed AEs, mainly fatigue (37%), dyspnoea (18%) and nausea (12%). AEs were present on 115/196 (59%) occasions on 300 mg/day and on 45/115 (39%) occasions on 200 mg/day (P<0.01). The mean level was 1.81+/-0.96 mg/L for patients reporting no AEs and 1.90+/-1.11 mg/L for those reporting AEs (P=not significant). Factors associated with AEs of grade >=2 were a daily dose of 300 versus 200mg (P=0.001) and asthma (P=0.008). Conclusion(s): A lower-than-recommended posaconazole tablet dose achieved therapeutic levels in most patients and was better tolerated. Males were more likely to achieve a therapeutic level. Underlying conditions affected the degree and frequency of AEs.Copyright © 2019 Oxford University Press. All rights reserved.

**Database:** EMBASE

**9. A cross-sectional survey to measure the prevalence of chronic pulmonary aspergillosis (CPA) complicating pulmonary tuberculosis in Northern Uganda**

**Author(s):** Page I.; Hosmane S.; Sawyer R.; Sharman A.; Onyachi N.; Opira C.; Opwonya J.; Richardson M.; Denning D.

**Source:** Open Forum Infectious Diseases; Sep 2017; vol. 4

**Publication Date:** Sep 2017

**Publication Type(s):** Conference Abstract

Available at [Open Forum Infectious Diseases](http://academic.oup.com/ofid/article/4/suppl_1/S721/4295817/ACrossSectional-Survey-to-Measure-the-Prevalence) - from Oxford Journals - Open Access

Available at [Open Forum Infectious Diseases](http://ovidsp.ovid.com/athens/ovidweb.cgi?T=JS&PAGE=fulltext&D=ovft&CSC=Y&NEWS=N&SEARCH=2328-8957.is+and+%224%22.vo+and+%22suppl_1%22.ip+and+%22S721%22.pg+or+%2210.1093/ofid/ofx163.1945%22.di) - from Ovid (Journals @ Ovid) - Remote Access

Available at [Open Forum Infectious Diseases](https://academic.oup.com/ofid/article-pdf/4/suppl_1/S721/20430212/ofx163.1945.pdf) - from Unpaywall

**Abstract:**Background. Chronic pulmonary aspergillosis (CPA) complicates pulmonary tuberculosis (TB). It has a 5-year mortality of up to 85%, but is treatable with itraconazole or surgery. The estimated global prevalence of CPA post-TB is 0.8-1.3 million cases. We conducted the first survey to measure the prevalence of CPA secondary to pulmonary TB. Methods. A cross-sectional survey of adults treated for pulmonary TB within the last 7 years in Gulu, Uganda. All underwent clinical assessment, chest X-ray and Aspergillusspecific IgG measurement by Siemens Immulite at a cut-off of 20mg/L, which has a sensitivity of 93% and a specificity of 98% for CPA diagnosis. Patients were resurveyed two years later. CT scan was performed in those with positive serology or chest X-ray signs of CPA. GeneXpert TB PCR testing was performed on those with productive cough. CPA was diagnosed in patients with ALL of the following; (1) >1 month cough or haemoptysis, (2) raised Aspergillus-specific IgG, and (3) paracavitary fibrosis, aspergilloma or progressive cavitation on imaging. Simple aspergilloma was diagnosed in asymptomatic patients with aspergilloma and positive serology. Results. In total, 400 patients were recruited between October 2012 and February 2013. 200 (50%) were HIV positive. Median age was 42 years (range 16-83). Thirtynine percent of patients were female. Median CD4 count in those with HIV was 415 cells/muL (range 0-1400). In total, 284 patients were re-surveyed between October 2014 and February 2015. Twenty-three (7.7%) of those resurveyed had raised Aspergillusspecific IgG levels. Twelve patients (4.2%) had CPA and 1 (0.4%) simple aspergilloma. A further three patients had a fungal ball on CT thorax, but normal levels of Aspergillusspecific IgG. HIV co-infection had no significant impact on the frequency of CPA. Three cases of recurrent pulmonary TB were identified, none in the CPA group. Conclusion. CPA complicates pulmonary tuberculosis. This data suggest the predicted global prevalence is accurate. This is a significant global public health problem, which is currently neglected. The clinical and radiological presentation of CPA is often identical to recurrent TB. In the absence of access to Aspergillus-specific IgG testing most cases of CPA are probably inaccurately diagnosed as recurrent ?smear-negative TB' and subjected to unnecessary and potentially toxic second-line TB therapy.

**Database:** EMBASE

**10. The Management of Chronic Pulmonary Aspergillosis: The UK National Aspergillosis Centre Approach**

**Author(s):** Maghrabi F.; Denning D.W.

**Source:** Current Fungal Infection Reports; Dec 2017; vol. 11 (no. 4); p. 242-251

**Publication Date:** Dec 2017

**Publication Type(s):** Review

Available at [Current fungal infection reports](https://link.springer.com/content/pdf/10.1007%2Fs12281-017-0304-7.pdf) - from Unpaywall

**Abstract:**Purpose of Review: Chronic pulmonary aspergillosis (CPA) is a serious long-term fungal disease of the lung with a worldwide prevalence. Treatment of CPA is not straightforward given the often-multiple associated co-morbidities, complex clinical picture, drug interactions, toxicities and intolerances. Recent Findings: First line treatment is oral itraconazole or voriconazole. In the event of intolerance or toxicity, patients may be swapped from itraconazole to voriconazole or vice versa. In the event of resistance or further intolerance, third line treatment with posaconazole could be initiated. In those with pan-azole resistance, short-term courses of intravenous liposomal amphotericin B or micafungin are fourth line therapy, keeping in mind the nephrotoxic effects of amphotericin B. Summary: The available evidence for current treatments in CPA is limited and based mostly on retrospective cohort studies. There is a real need to raise awareness of this devastating disease to enable early treatment as well as prospective drug trials and studies to identify potential patient factors that correlate with progression, severity and overall outcomes in order to target future therapies.Copyright © 2017, The Author(s).

**Database:** EMBASE

**11. Impact of liposomal amphotericin B therapy on chronic pulmonary aspergillosis**

**Author(s):** Newton P.J.; Harris C.; Denning D.W.; Morris J.

**Source:** Journal of Infection; Nov 2016; vol. 73 (no. 5); p. 485-495

**Publication Date:** Nov 2016

**Publication Type(s):** Article

**PubMedID:** 27373381

Available at [The Journal of infection](https://www.research.manchester.ac.uk/portal/files/50549376/AmBisome_therapy_for_DWD_FINAL_240516.pdf) - from Unpaywall

**Abstract:**Objectives To assess the clinical response and renal toxicity observed in chronic pulmonary aspergillosis (CPA) patients receiving >=1 short-courses of liposomal amphotericin (LAmB) (AmBisome) therapy. Methods A retrospective audit of clinical response and renal function was undertaken in 71 CPA patients (41 male) treated with LAmB at the National Aspergillosis Centre, including 20 patients receiving repeated treatment courses or long-term therapy (n = 5). Results Median age was 64 years (range 29-86 years). Treatment indications included respiratory symptoms (n = 33; 46.5%), constitutional symptoms (n = 2; 2.8%) or both (n = 36; 50.7%). 48 patients (73.8%) responded to their first LAmB course. Quality of life (QOL) improvements occurred in 37 (92.5%) of 40 patients with sufficient data available. Response rates for repeated short-courses of LAmB were 76.6%; QOL improvements were observed in 91.7% of treatment courses. All patients on long-term therapy demonstrated a response. 34 (50%) and 17 (25%) patients respectively developed an increased risk of acute kidney injury (AKI) or actual AKI with their first treatment; a significant reduction in geometric mean eGFR was observed and a similar pattern occurred following their second treatment course. Conclusions Whilst CPA is responsive to LAmB, caution should be exercised with repeated courses, if other treatments are available.Copyright © 2016

**Database:** EMBASE

**12. Long-term antifungal treatment improves health status in patients with chronic pulmonary aspergillosis: A longitudinal analysis**

**Author(s):** Al-Shair K.; Atherton G.T.; Harris C.; Ratcliffe L.; Newton P.J.; Denning D.W.

**Source:** Clinical Infectious Diseases; Sep 2013; vol. 57 (no. 6); p. 828-835

**Publication Date:** Sep 2013

**Publication Type(s):** Article

**PubMedID:** 23788240

Available at [Clinical infectious diseases : an official publication of the Infectious Diseases Society of America](https://academic.oup.com/cid/article-lookup/doi/10.1093/cid/cit411) - from Oxford Journals - Medicine

Available at [Clinical infectious diseases : an official publication of the Infectious Diseases Society of America](https://academic.oup.com/cid/article-pdf/57/6/828/17853305/cit411.pdf) - from Unpaywall

**Abstract:**Background. Chronic pulmonary aspergillosis (CPA) is an infectious disease that progressively destroys lung tissue. To date, no longitudinal data on the efficacy of antifungal treatment on health status in CPA patients exist.Methods. Using the standardized St George's Respiratory Questionnaire, the health status of 122 patients with was assessed at baseline and quarterly over 12 months. The score range was 0-100, where higher score indicates worse heath status, and a change of >=4 was deemed the minimal clinically important difference. Lung function, body mass index, Medical Research Council dyspnea scale, disease severity, and demographic data were reported.Results. Mean age of patients was 59 years, and 45% were female. Overall, patients with CPA had substantial health status impairment at baseline. After treatment, 47%-50% gained substantial health improvement with a mean reduction of score of 14 at both 6 and 12 months, whereas 32% deteriorated with a mean rise of score of 11 and 14 after 6 and 12 months of treatment and observation, respectively, and 21% were not much different (stable).Patients gained therapeutic benefit irrespective of their illness severity where >50% of those who had "poor" and "very poor" status at baseline improved with score reduction of >=4 after 6 months of treatment. Replicating this analysis using a health status category, we found that at least 50% of patients with a "poor/very poor" health status category at baseline improved significantly to "fair" or "good/very good" categories. Side effects burdened health status considerably. In multivariate analysis, dyspnea and disease severity significantly defined health status impairment.Conclusions. Antifungal therapy improved health status and prevented CPA progression in most patients. © 2013 The Author.

**Database:** EMBASE

**13. The correlation between serum trough levels and frequency of side effects of VRCZ at Nagasaki University Hospital, Nagasaki Japan**

**Author(s):** Hirano K.; Izumikawa K.; Ide S.; Iwanaga N.; Minematsu A.; Tashiro M.; Mihara T.; Kurihara S.; Nakamura S.; Imamura Y.; Miyazaki T.; Tsukamoto M.; Kakeya H.; Yamamoto Y.; Tashiro T.; Kohno S.; Hamada Y.; Takazono T.; Morinaga Y.; Yanagihara K.

**Source:** Mycoses; Jun 2012; vol. 55 ; p. 98

**Publication Date:** Jun 2012

**Publication Type(s):** Conference Abstract

Available at [Mycoses](https://go.openathens.net/redirector/nhs?url=https%3A%2F%2Fonlinelibrary.wiley.com%2Fdoi%2Ffull%2F10.1111%2Fj.1439-0507.2012.02206.x) - from Wiley Online Library Medicine and Nursing Collection 2019 - NHS

Available at [Mycoses](http://openurl.ebscohost.com/linksvc/linking.aspx?genre=article&issn=1439-0507&volume=55&spage=95&date=2012&title=Mycoses) - from EBSCO (MEDLINE Complete)

Available at [Mycoses](https://onlinelibrary.wiley.com/doi/pdfdirect/10.1111/j.1439-0507.2012.02206.x) - from Unpaywall

**Abstract:**Background: Fungal infection is one of the most serious problems in immunocompromised patients during the medication of anticancer and immunosuppressant drugs, and among HIV/AIDS patients. Although voriconazole (VRCZ) is a triazole antifungal agent with potent, broad spectrum, it may have some side effects, such as liver toxicity and visual disturbances. Therefore, the therapeutic drug monitoring (TDM) of VRCZ is quite important. In this study, we investigated the correlation between serum trough levels and frequency of side effects of VRCZ in the cases treated with VRCZ at Nagasaki University Hospital, Nagasaki, Japan. Objective and Method: Forty-eight cases with deep mycosis, treated with VRCZ under TDM at Nagasaki University Hospital from February 2009 to November 2010, were investigated retrospectively. We evaluated background of patients, serum trough levels and frequency of side effects related with VRCZ treatment. Result(s): A total of 117 serum samples were taken from patients during the indicated period. Seventy percent of cases were hematology patients, and the rest were respiratory, rheumatology and pediatrics patients. Patients of over 65 years occupied approximately 25% of cases. Underlying diseases and conditions were invasive pulmonary aspergillosis, chronic pulmonary aspergillosis, pulmonary cryptococcosis, candidemia and organ transplant patients with bone marrow. Thirty-seven and eighty cases were treated with intravenous VRCZ and oral VRCZ, respectively. Average durations of intravenous and oral VRCZ administration were 23.3 and 112.5 days, respectively. The mean serum trough of VRCZ was 2.83 mug ml-1 in oral administration and 3.85 mug ml-1 in intravenous administration. Major side effects were liver toxicity (15%) and visual disturbances (2.1%). Twenty-five percent of liver dysfunction cases were patients of over 65 years whose serum trough levels of VRCZ were relatively high value. The incidences of liver dysfunction were statistically higher in the patients whose serum trough levels were over 4.5 mug ml-1. Conclusion(s): Abnormal liver functions related with VRCZ administration were frequently seen in the patients with higher serum trough levels of VRCZ. As the serum trough levels of VRCZ have large individual diversity, TDM is strongly recommended for the patients who receive VRCZ treatment, especially in order individuals.

**Database:** EMBASE

**14. Chronic pulmonary aspergillosis (CPA): Role of <sup>18</sup>F -FDG PET/CT**

**Author(s):** Minniti L.; Popescu C.; Schiraldi G.; Chiericozzi M.; Nordin A.; Cabrini G.; Gay E.; Milella M.; Sara R.; Possa M.; Rossetti C.

**Source:** Respirology; Nov 2011; vol. 16 ; p. 59-60

**Publication Date:** Nov 2011

**Publication Type(s):** Conference Abstract

Available at [Respirology](https://go.openathens.net/redirector/nhs?url=https%3A%2F%2Fonlinelibrary.wiley.com%2Fdoi%2Ffull%2F10.1111%2Fj.1400-1843.2011.02071.x) - from Wiley Online Library Medicine and Nursing Collection 2019 - NHS

**Abstract:**Aim The spectrum of chronic pulmonary aspergillar infections (CPA) depends on the immunity conditions of the host (also as assumption of immunosuppressive drugs), the anatomical integrity, (previous lesions), functional alterations (bronchial obstruction), mucociliary clearance and environmental Aspergillus load. The diagnosis of CPA is difficult to make and is often delayed (by an average of months or years), contributing to increasing its morbidity and mortality. Different diagnostic strategies are currently being used, including radiology, cultures, several serologic tests and scintigraphic technique. The diagnosis is confirmed by demonstrating the invasion of the pulmonary tissue by septate hyphae typical of Aspergillus sp., and positive cultures of pulmonary tissue samples. This material is generally obtained through bronchoaspirate, transbronchial biopsy or transthoracic puncture, although these procedures have been reported to have a low yield. Also, scintigraphic techniques have been proposed including 67Ga-citrate and F-18 fluorodeoxyglucose positron emission scanning (18F-FDG PET/CT). The aim of our study was to evaluate the role of the 18F-FDG PET/CT in estimating the metabolic activity of the disease and in monitoring the response to the treatment. (Figure presented) Methods Thirty three cases of patients (19 males and 14 females, age range, 35-65 years), under treatment for aspergillosis infection, underwent PET/CT using 18F-FDG following standard protocol. All patients had serologic tests and radiological exams. Lung biopsies were performed on 3 of 33 patients and culture test for Aspergillus in sputum was executed on 8 of 33 patients. 18F-FDG PET/CT scan was repeated in 12 of 33 cases during the follow-up phase after 12-18 months. PET/CT images were analyzed by qualitative method using the background activity as index of normal uptake. PET findings were compared with serologic tests (identification of Aspergillus-specific IgE and IgG antibodies using immunofluorescence and radial immunodiffusion methods). Minor criteria for diagnosis included culture findings for Aspergillus in sputum (variable sensitivity) and lung biopsies (commonly requested in aggressive disease). PET findings were compared with serologic test for each patient and each study. Result(s): PET/CT findings were found positive in 27 of 33 patients (84%), with an agreement between PET and serologic test in 29/33 patients (82%) (25 positive and 4 negative). In one of the two cases of disagreement for PET positive / antibody negative, the culture test in sputum was found positive for A. Fumigatus. PET/CT findings at follow-up were found positive in 11 of 12 patients. PET and serologic test findings showed agreement in 7 of 11 patients. One single negative PET exam finding was confirmed by serologic test. In 13 of 27 patients with positive on PET scan, increased metabolic activity of the disease was found also on extrapulmonary sides. Conclusion(s): Our results indicate that 18F-FDG PET/CT scan is a reliable support tool for clinical and serological data in the diagnosis and follow-up of aspergillosis infection, providing additional information on metabolic activity, accurate anatomic localization and extent of disease. In the therapeutic field, the triazole antifungals (e.g. voriconazole) are currently a good option due to excellent efficacy, low toxicity and easy administration.

**Database:** EMBASE

**15. Efficacy and safety of posaconazole for chronic pulmonary aspergillosis**

**Author(s):** Felton T.W.; Baxter C.; Moore C.B.; Roberts S.A.; Hope W.W.; Denning D.W.

**Source:** Clinical Infectious Diseases; Dec 2010; vol. 51 (no. 12); p. 1383-1391

**Publication Date:** Dec 2010

**Publication Type(s):** Article

**PubMedID:** 21054179

Available at [Clinical infectious diseases : an official publication of the Infectious Diseases Society of America](https://academic.oup.com/cid/article-lookup/doi/10.1086/657306) - from Oxford Journals - Medicine

Available at [Clinical infectious diseases : an official publication of the Infectious Diseases Society of America](https://academic.oup.com/cid/article-pdf/51/12/1383/900388/51-12-1383.pdf) - from Unpaywall

**Abstract:**Background. Chronic pulmonary aspergillosis (CPA) is a severe, progressive respiratory infection characterized by multiple pulmonary cavities and increased levels of antibodies to Aspergillus species. We report the first use of posaconazole in patients with CPA. Methods. A retrospective study was performed. A composite clinical and radiological evaluation was used to assess response to posaconazole therapy. The rates of clinical response and failure after 6 and 12 months of therapy were determined. Kaplan-Meier survival models were developed to describe the time to clinical response and failure. The underlying diagnosis, the type of therapy (primary or salvage), Aspergillus antibody titer, and posaconazole serum concentrations were assessed as covariates. Aspergillus species were identified and minimum inhibitory concentrations (MICs) of triazoles were determined using standard techniques. Results. There were 79 patients that initially received posaconazole 400 mg twice per day. The median age of patients was 61 years, and 57% were male. Response to posaconazole was observed in 61% of patients at 6 months and in 46% at 12 months. Kaplan-Meier plots showed that the first response to posaconazole was observed in some patients only after approximately 1 year of therapy. Covariates were not significant. Adverse reactions were observed in 12 patients (15%) (nausea in 5, rash in 5, headache in 1, and lethargy in 1), leading to withdrawal of treatment for 9 patients. Aspergillus species were recovered from 22 patients. A posaconazole MIC of >8 mg/L was found in 4 isolates; in 1 of these isolates, this emerged during therapy. Treatment failed in all 4 patients from whom these 4 isolates had been recovered. Conclusion. Posaconazole is a safe and partially effective treatment for CPA. Prospective comparative studies are now required. © 2010 by the Infectious Diseases Society of America. All rights reserved.

**Database:** EMBASE

Strategy 850709

| **#** | **Database** | **Search term** | **Results** |
| --- | --- | --- | --- |
| 1 | Medline | ITRACONAZOLE/ | 5709 |
| 2 | Medline | (Itraconazole).ti,ab | 8638 |
| 3 | Medline | (voriconazole).ti,ab | 6037 |
| 4 | Medline | VORICONAZOLE/ | 3524 |
| 5 | Medline | (1 OR 2 OR 3 OR 4) | 14683 |
| 6 | Medline | ("chronic pulmonary aspergillosis" OR "chronic cavitary aspergillosis" OR "chronic fibrosing pulmonary aspergillosis" OR "simple aspergilloma").ti,ab | 351 |
| 7 | Medline | (5 AND 6) | 63 |
| 8 | Medline | (adverse ADJ3 (reaction* OR effect OR affect)).ti,ab | 80702 |
| 9 | Medline | (toxic*).ti,ab | 595525 |
| 10 | Medline | "DRUG-RELATED SIDE EFFECTS AND ADVERSE REACTIONS"/ | 31638 |
| 11 | Medline | ("side effect*").ti,ab | 241342 |
| 12 | Medline | (8 OR 9 OR 10 OR 11) | 894771 |
| 13 | Medline | (7 AND 12) | 10 |
| 14 | Medline | ("invasive aspergillosis" OR "allergic aspergillosis").ti,ab | 4133 |
| 15 | Medline | 13 not 14 | 9 |
| 16 | Medline | 15 [Languages English] [Humans] | 7 |
| 17 | EMBASE | ITRACONAZOLE/ | 29614 |
| 18 | EMBASE | (Itraconazole).ti,ab | 12024 |
| 19 | EMBASE | (voriconazole).ti,ab | 9392 |
| 20 | EMBASE | VORICONAZOLE/ | 19233 |
| 21 | EMBASE | (17 OR 18 OR 19 OR 20) | 42187 |
| 22 | EMBASE | ("chronic pulmonary aspergillosis" OR "chronic cavitary aspergillosis" OR "chronic fibrosing pulmonary aspergillosis" OR "simple aspergilloma").ti,ab | 516 |
| 23 | EMBASE | (21 AND 22) | 185 |
| 24 | EMBASE | (adverse ADJ3 (reaction* OR effect OR affect)).ti,ab | 120532 |
| 25 | EMBASE | (toxic*).ti,ab | 838624 |
| 26 | EMBASE | "DRUG-RELATED SIDE EFFECTS AND ADVERSE REACTIONS"/ | 170908 |
| 27 | EMBASE | ("side effect*").ti,ab | 360189 |
| 28 | EMBASE | (24 OR 25 OR 26 OR 27) | 1373284 |
| 29 | EMBASE | (23 AND 28) | 21 |
| 30 | EMBASE | ("invasive aspergillosis" OR "allergic aspergillosis").ti,ab | 5903 |
| 31 | EMBASE | 29 not 30 | 18 |
| 32 | EMBASE | 31 [English language] [Humans] | 17 |
