# Supplementary material for: Tolerability of oral itraconazole and voriconazole for the treatment of chronic pulmonary aspergillosis: A systematic review and meta-analysis
Source: PLoS One. 2020 Oct 14;15(10):e0240374. doi: 10.1371/journal.pone.0240374 (PMC7556473; doi:10.1371/journal.pone.0240374)
Supplement: S3 File — (DOCX) [file pone.0240374.s005.docx]

**Risk of Bias Assessment:** Tolerability of Oral Itraconazole and Voriconazole for The Treatment of Chronic Pulmonary Aspergillosis: A Systematic Review and Meta-Analysis.

All the studies included were independently assessed for risk of bias. Randomized clinical trials were assessed using Risk of Bias, RoB 2 tool (Cochrane Collaboration [1]). Risk of bias was rated using in-built algorithm as “low”, “some concerns” and “high”. Observational studies were assessed using Newcastle–Ottawa Scale (NOS) for evaluation of cohort studies [2]. NOS assesses for three dimensions of a study; sample selection (3 points), comparability (2 points) and the outcome of the study (4 points) with a total of 9 points. Quality of the studies was graded as good, fair or poor by awarding stars in each parameter following the guidelines of the NOS. Five studies [3-7] were graded as ‘good quality’ and three studies [8-10] as of ‘fair quality’ using NOS for risk of bias assessment.

**Randomised Clinical Trial (RoB 2 Tool[1]).**

| **Unique ID** | 1 | **Study ID** | RCT001 | **Assessor** | Ronald Olum & Felix Bongomin |
| --- | --- | --- | --- | --- | --- |
| **Ref or Label** | Agarwal et al 2013 [11] | **Aim** | assignment to intervention (the 'intention-to-treat' effect) |  |  |
| **Experimental** | Itraconazole | **Comparator** | Supportive | **Source** |  |
| **Outcome** | Clinical, radiological and overall response | **Results** | Frequency and quantity of symptoms; 50% reduction in the diameter of lesions. | **Weight** | 1 |
| **Domain** | **Signalling question** | | | **Response** | **Comments** |
| **Bias arising from the randomization process** | 1.1 Was the allocation sequence random? | | | Y | The subjects were randomly assigned to either the control arm (supportive therapy alone) or the itraconazole arm (itraconazole 400 mg day with supportive therapy). The randomisation sequence was computer generated using the statistical package Stats-Direct for MS-Windows (Version 2.7.2, England, Stats- Direct Ltd, 2005. http://www.statsdirect.com). The assignments were placed in sealed opaque envelopes and each patient’s assignment to a particular group was made sequentially. Blinding of treatment allocation was not possible. |
|  | 1.2 Was the allocation sequence concealed until participants were enrolled and assigned to interventions? | | | Y |  |
|  | 1.3 Did baseline differences between intervention groups suggest a problem with the randomization process? | | | N |  |
|  | **Risk of bias judgement** | | | **Low** |  |
| **Bias due to deviations from intended interventions** | 2.1. Were participants aware of their assigned intervention during the trial? | | | Y | Blinding of treatment allocation was not possible. |
|  | 2.2. Were carers and people delivering the interventions aware of participants' assigned intervention during the trial? | | | Y |  |
|  | 2.3. If Y/PY/NI to 2.1 or 2.2: Were there deviations from the intended intervention that arose because of the experimental context? | | | PN | Adherence to itraconazole was assessed by instructing patient to bring the empty pill covers of the drug. |
|  | 2.4 If Y/PY to 2.3: Were these deviations likely to have affected the outcome? | | | NA |  |
|  | 2.5. If Y/PY/NI to 2.4: Were these deviations from intended intervention balanced between groups? | | | NA |  |
|  | 2.6 Was an appropriate analysis used to estimate the effect of assignment to intervention? | | | NI |  |
|  | 2.7 If N/PN/NI to 2.6: Was there potential for a substantial impact (on the result) of the failure to analyse participants in the group to which they were randomized? | | | PN |  |
|  | **Risk of bias judgement** | | | **Some concerns** |  |
| **Bias due to missing outcome data** | 3.1 Were data for this outcome available for all, or nearly all, participants randomized? | | | Y |  |
|  | 3.2 If N/PN/NI to 3.1: Is there evidence that result was not biased by missing outcome data? | | | NA |  |
|  | 3.3 If N/PN to 3.2: Could missingness in the outcome depend on its true value? | | | NA |  |
|  | 3.4 If Y/PY/NI to 3.3: Is it likely that missingness in the outcome depended on its true value? | | | NA |  |
|  | **Risk of bias judgement** | | | **Low** |  |
| **Bias in measurement of the outcome** | 4.1 Was the method of measuring the outcome inappropriate? | | | N |  |
|  | 4.2 Could measurement or ascertainment of the outcome have differed between intervention groups? | | | N |  |
|  | 4.3 Were outcome assessors aware of the intervention received by study participants? | | | PY |  |
|  | 4.4 If Y/PY/NI to 4.3: Could assessment of the outcome have been influenced by knowledge of intervention received? | | | PN |  |
|  | 4.5 If Y/PY/NI to 4.4: Is it likely that assessment of the outcome was influenced by knowledge of intervention received? | | | NA |  |
|  | **Risk of bias judgement** | | | **Low** |  |
| **Bias in selection of the reported result** | 5.1 Were the data that produced this result analysed in accordance with a pre-specified analysis plan that was finalized before unblinded outcome data were available for analysis? | | | PN | According to the protocol on https://clinicaltrials.gov/ct2/show/NCT01259336, the following criteria for radiological response was set: Complete response- It is defined as complete disappearance of the aspergilloma. Partial response- It is defined as 30% decrease in the sum of the longest diameters of all the lesions. Progressive disease- It is defined as appearance of any new lesions or >20% increase in the sum of the longest diameters of all measurable lesions. In the final analyses, 50% was used as a criterion for improvement. |
|  | 5.2 ... multiple eligible outcome measurements (e.g. scales, definitions, time points) within the outcome domain? | | | N |  |
|  | 5.3 ... multiple eligible analyses of the data? | | | N |  |
|  | **Risk of bias judgement** | | | **Low** |  |
| **Overall bias** | **Risk of bias judgement** | | | **Some concerns** | The study was open label with no blinding and therefore has some risk of bias. |

**Observational Studies**

| **Parameter** | **Selection** | | | | **Comparability** | **Outcome** | | | **Quality Score** |
| --- | --- | --- | --- | --- | --- | --- | --- | --- | --- |
| **Question** | Representativeness of the exposed cohort | Selection of the non-exposed cohort | Ascertainment of exposure | Demonstration that outcome of interest was not present at start of study | Comparability of cohorts on the basis of the design or analysis | Assessment of outcome | Was follow-up long enough for outcomes to occur | Adequacy of follow up of cohorts |  |
| Bongomin (2019)[3] | * | NA | * | * | ** | * | 12 months * | * | Good |
| Bongomin (2018)[4] | 92% (206/233) of all patients with CPA in 2 years at NAC were included * | NA | * | * | ** | * | 12 months * | * | Good |
| Cucchetto (2015)[6] | All patients refered to the hospital meeting the eligibility criteria * | NA | * | * | - | * | 12 months * | * | Good |
| Al-Shair (2013)[5] | Large sample size (122 patients) referred in 3 years to NAC * | NA | * | * | - | * | 6-12 months * | * | Good |
| Cadranel (2012)[9] | Patients from 18 hospitals over a 3-year period * | NA | * | * | - | * | 6-18 months * | * | Fair |
| Saito (2012)[7] | CPA patients were eligible fo voriconazole recruited from multiple centres * | NA | * | * | * | * | 12 weeks * | * | Good |
| Camuset (2007)[8] | All eligible patients in 12 teaching hospitals were included * | NA | * | * | - | * | Median: 10 months * | * | Fair |
| Jain (2005)[10] | All patients reffered to the Prof Denning until 2004. * | NA | * | * | - | * | 3-12 months * | * | Fair |

**References**

1. Sterne JAC, Savović J, Page MJ, Elbers RG, Blencowe NS, Boutron I, et al. RoB 2: a revised tool for assessing risk of bias in randomised trials. bmj; 2019;366.

2. Wells G, Shea B, O’Connell D, Peterson J, Welch V, Losos M, et al. Newcastle-Ottawa quality assessment scale cohort studies. Ontario, Canada: University of Ottawa; 2014.

3. Bongomin F, Maguire N, Moore CB, Felton T, Rautemaa-Richardson R. Isavuconazole and voriconazole for the treatment of chronic pulmonary aspergillosis: A retrospective comparison of rates of adverse events. Mycoses; 2019;62(3):217-22. Epub 2018/12/21. doi: 10.1111/myc.12885. PubMed PMID: 30570179.

4. Bongomin F, Harris C, Hayes G, Kosmidis C, Denning DW. Twelve-month clinical outcomes of 206 patients with chronic pulmonary aspergillosis. PLoS One; 2018;13(4):e0193732. Epub 2018/04/11. doi: 10.1371/journal.pone.0193732. PubMed PMID: 29634721; PubMed Central PMCID: PMCPMC5892866.

5. Al-Shair K, Atherton GT, Harris C, Ratcliffe L, Newton PJ, Denning DW. Long-term antifungal treatment improves health status in patients with chronic pulmonary aspergillosis: a longitudinal analysis. Clin Infect Dis; 2013;57(6):828-35. Epub 2013/06/22. doi: 10.1093/cid/cit411. PubMed PMID: 23788240; PubMed Central PMCID: PMCPMC3749749.

6. Cucchetto G, Cazzadori A, Conti M, Cascio GL, Braggio P, Concia E. Treatment of chronic pulmonary aspergillosis with voriconazole: review of a case series. Infection; 2015;43(3):277-86. Epub 2014/11/30. doi: 10.1007/s15010-014-0711-4. PubMed PMID: 25432571.

7. Saito T, Fujiuchi S, Tao Y, Sasaki Y, Ogawa K, Suzuki K, et al. Efficacy and safety of voriconazole in the treatment of chronic pulmonary aspergillosis: experience in Japan. Infection; 2012;40(6):661-7. Epub 2012/09/08. doi: 10.1007/s15010-012-0322-x. PubMed PMID: 22956473.

8. Camuset J, Nunes H, Dombret MC, Bergeron A, Henno P, Philippe B, et al. Treatment of chronic pulmonary aspergillosis by voriconazole in nonimmunocompromised patients. Chest; 2007;131(5):1435-41. Epub 2007/04/03. doi: 10.1378/chest.06-2441. PubMed PMID: 17400661.

9. Cadranel J, Philippe B, Hennequin C, Bergeron A, Bergot E, Bourdin A, et al. Voriconazole for chronic pulmonary aspergillosis: a prospective multicenter trial. Eur J Clin Microbiol Infect Dis; 2012;31(11):3231-9. Epub 2012/07/12. doi: 10.1007/s10096-012-1690-y. PubMed PMID: 22782438; PubMed Central PMCID: PMCPMC3479377.

10. Jain LR, Denning DW. The efficacy and tolerability of voriconazole in the treatment of chronic cavitary pulmonary aspergillosis. J Infect; 2006;52(5):e133-7. Epub 2006/01/24. doi: 10.1016/j.jinf.2005.08.022. PubMed PMID: 16427702.

11. Agarwal R, Vishwanath G, Aggarwal AN, Garg M, Gupta D, Chakrabarti A. Itraconazole in chronic cavitary pulmonary aspergillosis: a randomised controlled trial and systematic review of literature. Mycoses; 2013;56(5):559-70. Epub 2013/03/19. doi: 10.1111/myc.12075. PubMed PMID: 23496375.
