# Supplementary material for: Tolerability of oral itraconazole and voriconazole for the treatment of chronic pulmonary aspergillosis: A systematic review and meta-analysis
Source: PLoS One. 2020 Oct 14;15(10):e0240374. doi: 10.1371/journal.pone.0240374 (PMC7556473; doi:10.1371/journal.pone.0240374)
Supplement: Attachment — Submitted filename: report PONE-D-20-17016.docx [file pone.0240374.s006.docx]

Reviewer report on the manuscript PONE-D-20-17016_titled **"** **Tolerability of Oral Itraconazole and Voriconazole for The Treatment of Chronic Pulmonary Aspergillosis: A Systematic Review and Meta-Analysis**". The present research was aimed to evaluate the frequency of adverse events of itraconazole and voriconazole for the treatment of CPA. I have some major limitations that that should be considered for considering publication of this manuscript, these include:

1. **In the abstract**
   a. **(line 37-38) the sentence started with" We included 10 eligible studies, in 8 studies, 366 patients were treated with voriconazole (69%) and 168 with itraconazole (31%) in 2 studies" was ended with 2 studies???.. it should be rephrased to be understandable. I understand that, the 366 patients were enrolled in the 8 studies but what about the two other studies???? What were the differences between the 8 and the 2 studies?**
   b. **Lines 40 and 42, the authors should include the number of patients between brackets adjacent to the 36% and 25%.**
   c. **Lines 42 and 43, the author should not start the sentence with numbers and both sentences should sentences should be lined to avoid repetition of verbs.**
   d. **The whole abstract should be rephrased particularly the % and (number of patients) in order to presented in more clearly and understandable way.**
2. **In the introduction**
   a. **"line 77" the sentence started with "Few studies have reported adverse events …….etc) however, the authors did not make citation, therefore, the author should inserted the appropriate citation for these few studies.**
   b. **Abbreviations should be defined at first mention and used consistently thereafter. In line 77, the author should replace adverse events with the abbreviation (AEs) as previously indicated.**
   c. **There are a lot of relevant literature are available and I recommend the authors to be include them in both introduction and discussion sections, examples**
      i. Bongomin F, Asio LG, Baluku JB, Kwizera R, Denning DW. Chronic Pulmonary Aspergillosis: Notes for a Clinician in a Resource-Limited Setting Where There Is No Mycologist. *J Fungi (Basel)*. 2020;6(2):75. Published 2020 Jun 2. doi:10.3390/jof6020075**.**
      ii. Jenks JD, Hoenigl M. Treatment of Aspergillosis. *J Fungi (Basel)*. 2018;4(3):98. Published 2018 Aug 19. doi:10.3390/jof4030098
      iii. Alastruey-Izquierdo A, Cadranel J, Flick H, et al. Treatment of Chronic Pulmonary Aspergillosis: Current Standards and Future Perspectives. *Respiration*. 2018;96(2):159-170. doi:10.1159/000489474
      iv. Maghrabi F, Denning DW. The Management of Chronic Pulmonary Aspergillosis: The UK National Aspergillosis Centre Approach. *Curr Fungal Infect Rep*. 2017;11(4):242-251. doi:10.1007/s12281-017-0304-7
      v. Baxter CG, Marshall A, Roberts M, Felton TW, Denning DW. Peripheral neuropathy in patients on long-term triazole antifungal therapy. *J Antimicrob Chemother*. 2011;66(9):2136-2139. doi:10.1093/jac/dkr233
      vi. Sambatakou H, Dupont B, Lode H, Denning DW. Voriconazole treatment for subacute invasive and chronic pulmonary aspergillosis. *Am J Med*. 2006;119(6):527.e17-527.e5.27E24. doi:10.1016/j.amjmed.2005.11.028
3. **Type of article should be changed from" Research article" to "review article) since it was meta-analysis and in silico study**

Therefore, and according to the above mentioned remarks I advised major revision of this manuscript under its current status.
